# Anesthesia for Urgent Gastroscopy in Cold Agglutinin Disease: A Case Report and Literature Review

**DOI:** 10.7759/cureus.84607

**Published:** 2025-05-22

**Authors:** Addison H Zhang, Cassie L Dow, Jack Bellamy

**Affiliations:** 1 Anaesthesiology, Rockhampton Hospital, Rockhampton, AUS; 2 Medicine, University of New South Wales, Kensington, AUS; 3 Anaesthesiology, Campbelltown Hospital, Campbelltown, AUS

**Keywords:** anaesthesiology, cold agglutinin, git endoscopy, hemolysis, upper gi bleeding

## Abstract

We present the case of a 76-year-old lady who underwent an emergency gastroscopy for suspected upper gastrointestinal bleeding on a background of cold agglutinin disease, a rare autoimmune haemolytic anaemia triggered by cold temperatures. Several pre-operative and intra-operative precautions were taken to minimise the risk of hypothermia. Sedation anaesthesia was used with midazolam and gradual titration of propofol with high-flow nasal cannulae to maintain oxygenation. The anaesthetic proceeded safely and without complications, and we compared our technique to the literature.

## Introduction

Cold agglutinin disease (CAD) is a rare form of autoimmune haemolytic anaemia (AIHA), with an overall incidence of 1 in 1,000,000 per annum. It mainly develops in the sixth and seventh decades of life; however, it may rarely affect those younger than 30 years old [1]. The disease is generally an IgM-mediated process, with the hallmark feature of activation from cold temperatures. These autoantibodies are directed against I/i carbohydrate antigens located on the red blood cell (RBC) surface, causing agglutination and lysis. In addition to haemolysis, clinical manifestations include circulatory symptoms such as light-headedness, dyspnoea, and diaphoresis, as well as cutaneous manifestations such as Raynaud’s phenomenon, acrocyanosis, and livedo reticularis. Treatment options include steroids and immunotherapy; however, complete and sustained remissions are uncommon, and the mainstay of management is avoidance of cold temperatures [2]. Anaesthesia causes impairment of thermoregulation and hypothermia that may cause significant morbidity in patients with CAD [3]. Here, we discuss the multidisciplinary management of a patient with a history of CAD who underwent an urgent gastroscopy for suspected upper gastrointestinal bleeding. Informed written consent was gained from the patient to publish this report.

## Case presentation

A 76-year-old lady presented to the hospital complaining of extreme lethargy over several weeks, associated with intermittent black stools, on a background of CAD and active anticoagulation following a pulmonary embolism (PE) diagnosed four months prior. In addition, she had a history of recent upper respiratory tract infection (URTI), Sjogren’s syndrome, chronic kidney disease (CKD), hypertension, iron deficiency, diverticulosis, gastrointestinal reflux disease and depression.

Blood tests revealed a haemoglobin level of 58g/L, a significant drop from 85g/L tested two weeks prior. The white cell count was 6.4x10^9/L, and the platelets were 491x10^9/L. Electrolytes and liver function tests were within normal range. Her kidney function was at baseline with creatinine 122umol/L and urea 11.4mmol/L. A haemolysis screen was negative with bilirubin level 12umol/L, haptoglobin 1.24g/L, reticulocyte counts 52x10^9/L and lactate dehydrogenase 177U/L. These laboratory results are summarised in Table 1. The direct antiglobulin test was positive; however, the patient had a known history of RBC antibodies. Examination did not reveal any active bleeding rectally. The rapid drop in haemoglobin, suspected melaena, and active anticoagulation were concerning for an upper gastrointestinal bleed. As a result, the patient was transfused with three units of packed red blood cells and admitted for a planned endoscopy.

**Table 1:** Selected laboratory results for the patient on admission, showing significant anaemia, stable CKD, and no active haemolysis. Reference ranges adapted from the RCPA: The Royal College of Pathologists of Australasia CKD: Chronic kidney disease

Pathology (units)	Result	Reference Range (RCPA)
Haemoglobin (g/L)	58	>120
White cell count (10^9^/L)	6.4	3.5 – 11.0
Platelets (10^9^/L)	491	150 – 400
Creatinine (mol/L)	122	45 – 90
Urea (mmol/L)	11.4	3.0 – 10.0
Bilirubin (µmol/L)	12	1 – 20
Haptoglobin (g/L)	1.24	0.3 – 2.0
Reticulocyte count (10^9^/L)	52	23 – 100
Lactate dehydrogenase (U/L)	177	120 – 250

Given the complex comorbidities, multi-disciplinary team input was sought involving haematology, anaesthesia and gastroenterology. A consensus decision was reached to proceed with gastroscopy, with precautions to avoid hypothermia. Prior to the case, the theatre was warmed to 24°C, the maximum setting possible. A fluid and forced air warmer was prepared, and cross-matched red cells were prepared by the blood bank. High-flow nasal cannulae were used to deliver oxygen. The team arranged warmed irrigation fluid (37°C) and requested warmed insufflation gas; however, this was not available at our institution.

Sedation anaesthesia was achieved with 1mg of midazolam and propofol delivered via a Schnider target-controlled infusion (TCI) pump, with gradual titration until loss of response to painful stimuli, while maintaining spontaneous ventilation. This occurred at an effect site concentration of 3mcg/ml. Furthermore, the oropharynx was topicalised with five sprays of warmed 10% lignocaine, and the oral mucosa was moistened with warm medical water to facilitate safe passage of the scope of her background of Sjögren’s syndrome.

**Figure 1:**
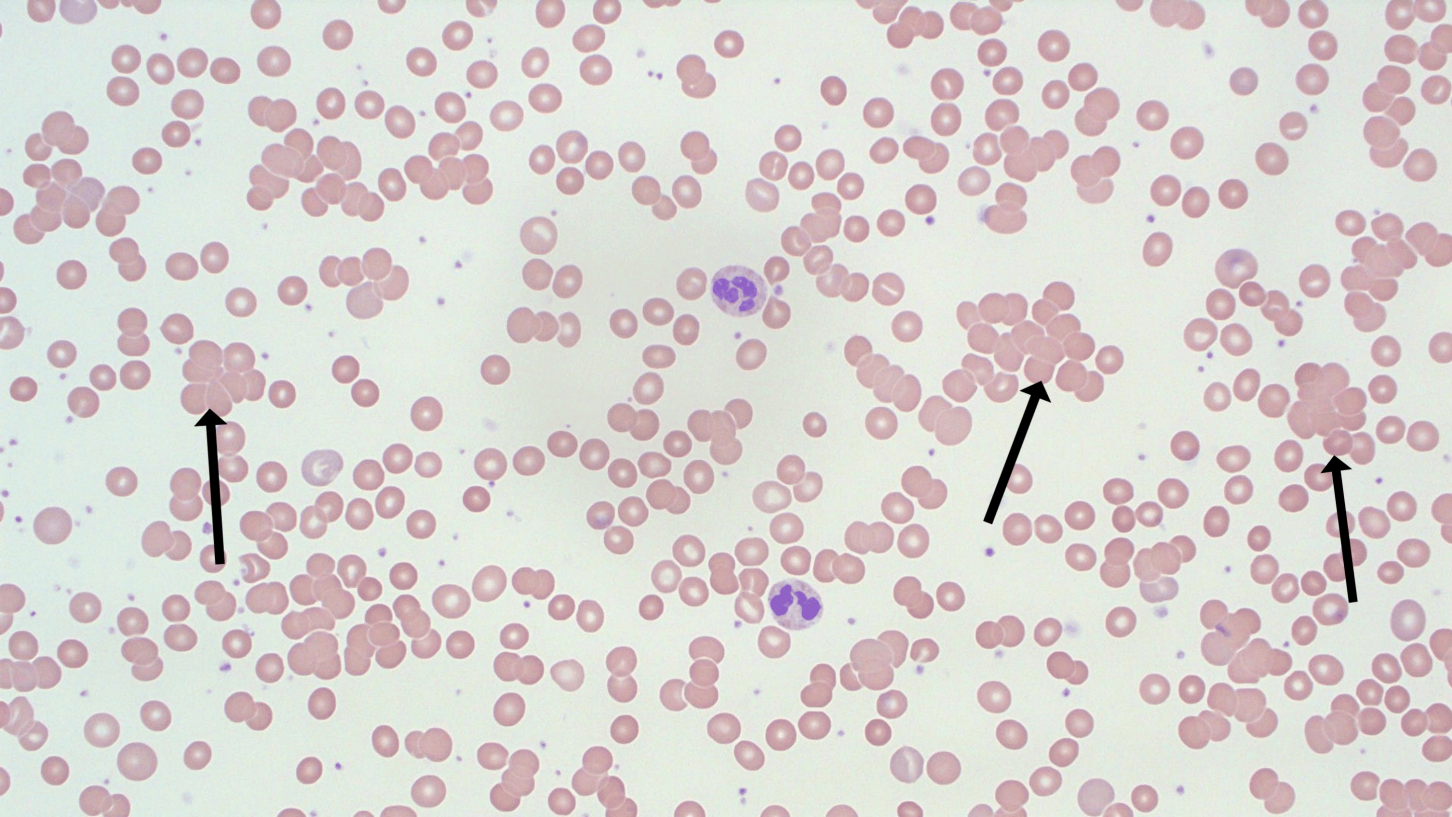
Previous blood film of the patient (40x objective) showing multiple sites of red cell agglutination (arrows) and mild polychromasia secondary to an active flare of CAD CAD: Cold agglutinin disease

Gastroscopy revealed no active bleeding in the oesophagus, stomach and duodenum. The patient had stable haemodynamic and respiratory parameters throughout the case. The pre-operative surface temperature of the patient was 37.0°C (tympanic), and at the end of the case, it was 36.1°C. Ongoing monitoring the following day showed a stable haemoglobin of 88g/L and no evidence of cold agglutinin disease or haemolysis. She was discharged from the hospital with plans for a haematology clinic follow-up and colonoscopy. Figure 1 shows the blood film of the patient.

## Discussion

Endoscopic procedures are one of the most common reasons for requiring sedation; however, they pose a similar risk of perioperative hypothermia due to exposure to a cool environment and drug-induced impaired thermoregulation. This results in vasodilation and reduction in metabolic rate, as well as inhibition of normal thermoregulatory measures such as vasocontraction and shivering. Although several case reports have described the anaesthetic management of CAD in the context of major cardiovascular, colorectal, urological, and orthopaedic surgery [4-8], to our knowledge this is the first detailed description of management in an urgent endoscopic case.

Several precautions were taken to minimise the risk of the procedure, summarised in Table 2. First, close consultation was sought with the haematology and procedural teams pre-operatively, and a multidisciplinary team meeting, including theatre staff, was held immediately prior to the case. We focused our anaesthetic on minimising the risk of hypothermia through increasing theatre temperature, active warming, and using warmed infusions and irrigation fluid. These measures have previously been described in the literature for patients with CAD undergoing general anaesthesia for major laparoscopic and abdominal surgeries [6-8]. In this case, however, we elected to use sedation because the patient fasted, had no history of vomiting or haematemesis, and we wanted to avoid the greater distributive heat loss associated with general anaesthesia. Furthermore, we believed spontaneous ventilation was safer given the history of PE and recent URTI. High-flow nasal cannulae (Optiflow THRIVE - Fisher and Paykel) were used accordingly to deliver heated and humidified oxygen to reduce thermal losses from the airway.

**Table 2:** Perioperative recommendations in endoscopy with cold agglutinin disease

Phases	Management strategies
Pre-operative	- Multidisciplinary team input involving haematology, surgery / proceduralists, theatre staff and anaesthetics - Encourage patient to remain warm - Consider medical treatments or plasmapheresis to reduce cold agglutinin titre
Intraoperative	- Raise operating room temperature - Warming with blankets and forced air devices - Ensure warmed blood products available - High flow nasal cannula or warmed and humidified ventilator circuit - Warm intravenous and surgical fluids - Warm insufflation gas if available - Consider continuous temperature and urine monitoring if prolonged - Consider amino acid infusion
Postoperative	- Ongoing active warming as required - Monitor for CAD with blood results and clinical symptoms and signs - Daily blood investigations: Fully blood count (FBC), Electrolytes, Urea, Creatinine (EUC), haptoglobin, lactose dehydrogenase, bilirubin - Inpatient haematology consultation and close outpatient follow-up

Nonetheless, several unique challenges were encountered in the management of this patient. We considered previous reports which describe assessing anti-I titres and, if high (>1:64), utilising plasmapheresis to remove antibodies pre-operatively [6]. The last measured titres (1:128) in this patient were from several years ago but given the urgent nature of the case and in consultation with the haematology team, we concluded that plasmapheresis would not be feasible due to time constraints. Amino acid infusions have also been described to increase heat production from enhanced metabolism and have been successfully used in the management of perioperative CAD patients, but this was not available at our institution on short notice [9]. Other treatments for CAD, including intravenous immunoglobulin (IVIG), immunomodulators, or steroids, were considered, but in the acute context, there was no strong evidence it would reduce the risk of AIHA, and steroids may have exacerbated the suspected upper GI bleeding [2].

Invasive temperature monitoring was not used, as the case was relatively short; nasal and oesophageal probes were not feasible due to equipment needs, and urinary probes would expose the patient to infection risk. Instead, pre- and post-operative temperatures were taken, which did not show any significant hypothermia, and indeed, no clinical or biochemical indicators of CAD or AIHA were found in follow-up care.

## Conclusions

We demonstrate that multimodal warming strategies and utilisation of high-flow nasal prongs provide a safe sedation anaesthetic for CAD patients undergoing short, high-risk procedures such as endoscopy for suspected upper GI bleeding. This provides a practical alternative to previous literature that describes techniques for CAD patients undergoing major procedures under general anaesthesia.
